# Enthesitis indices identify different patients with this characteristic in axial and peripheral spondyloarthritis and also in psoriatic arthritis: ASAS-PerSpA data

**DOI:** 10.1186/s13075-023-03080-0

**Published:** 2023-06-08

**Authors:** Raquel Ena María Granados, M. Lourdes Ladehesa-Pineda, M. Ángeles Puche-Larrubia, Alejandro Escudero-Contreras, Maxime Dougados, Eduardo Collantes-Estevez, Clementina López-Medina

**Affiliations:** 1 Rheumatology Department, Reina Sofia University Hospital, Cordoba, Spain; 2 Maimonides Biomedical Research Institute of Cordoba (IMIBIC), Cordoba, Spain; 3 University of Cordoba, Cordoba, Spain; 4 Rheumatology Department, Cochin Hospital, AP-HP, Paris, FR. INSERM U1153, CRESS, Université Paris-Cité, Paris, France

**Keywords:** Enthesitis, Indices, Spondyloarthritis, Psoriatic arthritis

## Abstract

**Background:**

In axial spondyloarthritis (axSpA), peripheral SpA (pSpA) and psoriatic arthritis (PsA), enthesitis is a hallmark clinical feature that can be assessed by the SPARCC index, LEI, MASES and MEI. These indices evaluate different locations, which may identify different numbers of patients with enthesitis among SpA subtypes. Thus, the aim of this study was to evaluate whether the proportion of patients with at least one enthesitis across these three most prevalent SpA subtypes differs according to the index used and to evaluate the level of agreement among indices in detecting patients with enthesitis.

**Methods:**

A total of 4185 patients (2719 axSpA, 433 pSpA and 1033 PsA) from the international and cross-sectional ASAS-PerSpA study were included. The proportion of patients with enthesitis identified by the indices was evaluated across the three diseases. Pairwise agreement between indices was computed using Cohen’s kappa.

**Results:**

The prevalence rates of patients with at least one enthesitis according to the MEI, MASES, SPARCC index and LEI were 17.2%, 13.5%, 10.7%, and 8.3%, respectively. In axSpA, the indices that identified the most patients with enthesitis were the MEI and MASES (98.7% and 82.4%, respectively); in pSpA and PsA, the indices that identified the most patients with enthesitis were the MEI and SPARCC index (MEI: 100% and SPARCC: 84.6%; MEI: 97.3% and SPARCC: 77%, respectively). In the total population, the MASES vs. MEI showed the strongest agreement (absolute agreement 96.3%; kappa: 0.86); similar results were obtained in axSpA patients (97.3%; 0.90). In pSpA and PsA patients, the SPARCC vs. MEI (97.2%; 0.90 and 95.4%; 0.83, respectively) showed the strongest agreement.

**Conclusions:**

These results suggest that the prevalence of patients with enthesitis across SpA subtypes differs depending on the disease and the index used. The MEI and MASES appeared best for assessing enthesis in SpA and axSpA, while the MEI and SPARCC index appeared best for assessing enthesitis in pSpA and PsA.

## Key messages

1. The prevalence of enthesitis differs depending on the disease and index used.
2. The MEI and MASES appeared best for assessing enthesis in axSpA patients.
3. The MEI and SPARCC appeared best for assessing enthesitis in pSpA and PsA patients.

## Introduction

Spondyloarthritis (SpA) represents a group of rheumatic inflammatory diseases characterized by the involvement of the spine and sacroiliac joints. These entities have been classified by the Assessment of Spondyloarthritis International Society (ASAS) [1, 2] according to their clinical presentation as predominantly axial spondyloarthritis (axSpA) or predominantly peripheral spondyloarthritis (pSpA) [3]. Psoriatic arthritis (PsA) represents a particular subtype of SpA that is characterized by chronic inflammatory arthritis in patients with a personal or familial history of psoriasis [4]. Patients with SpA and PsA may suffer from peripheral musculoskeletal manifestations such as peripheral arthritis, dactylitis and enthesitis [5].

Enthesitis is an important and frequent manifestation in patients with SpA and PsA. In recent years, it has received increasing attention, as SpA (both axSpA and pSpA) and PsA share this phenomenon as a hallmark clinical feature. An enthesis is the insertion of tendons and ligaments into the bone surface. These sites are usually located outside the joint, providing transduction of mechanical forces and stability. In the human body, there are more than a hundred entheses. Enthesitis can result from repeated mechanical overloading but is also a clinical feature in SpA and PsA, triggered predominantly by an innate immune response [6].

Enthesitis is part of the entry items of the ASAS classification criteria for peripheral SpA [2]. It has been shown that enthesitis is associated with higher disease activity and a worse quality of life in patients with SpA [7].

To evaluate this feature in observational studies, clinical indices have been developed: the Mander Enthesitis Index (MEI) [8], the Spondyloarthritis Research Consortium of Canada (SPARCC) Enthesitis Index [9], the Leeds Enthesitis Index (LEI) [10], and the Maastricht Ankylosing Spondylitis Enthesitis Score (MASES) [11]. However, there are no recommendations regarding the use of a specific index among the SpA subtypes. In addition, they may identify different numbers of patients with enthesitis among SpA subtypes, as these indices target different enthesis locations.

The aims of this study were (a) to describe the individual locations of enthesitis in patients with axSpA, pSpA and PsA; (b) to evaluate whether the prevalence of patients with at least one enthesitis across the three SpA subtypes differs depending on the index used (the SPARCC index, LEI, MASES or MEI); and (c) to evaluate the level of agreement among these indices for identifying patients with at least one enthesitis in the axSpA, pSpA and PsA populations.

## Methods

### Design

The present study is an ancillary analysis of data from the international and cross-sectional ASAS-PerSpA study that included 24 participating countries [3]. Both the design and patient recruitment have been described elsewhere [3].

### Patients

In this ancillary analysis, we included adult patients diagnosed with axSpA, PsA or pSpA by a rheumatologist. Patients had to be capable of understanding and completing questionnaires. The present study was developed according to the guidelines of Good Clinical Practice, described in the original study [3]. It was also approved by the ethics committees of each country, and written informed consent was obtained from all subjects.

### Assessment of enthesitis

The presence of enthesitis during the study visit was determined using a specific case report form (CRF). This CRF included information on 36 enthesis locations in which the investigator reported the level of tenderness from 0, 1, 2 and 3 (no pain, mild tenderness, moderate tenderness, wince/withdraw, respectively). A positive enthesitis at any location was considered if the level of tenderness was > 1. With this CRF, the MEI, SPARCC index, LEI and MASES could be computed in the same patient. Only the 7^th^ costochondral joint was missing in this CRF, slightly affecting the evaluation of the MASES, since this is a very infrequent location of enthesitis.

- The MEI involves the assessment of 66 entheses [8]. This index includes both axial and peripheral locations: the ischial tuberosities, posterior superior iliac spines, plantar fascia, insertion of the Achilles tendon, medial and lateral condyles of the femur, greater trochanter of the femur, anterior superior iliac spines, iliac crests, medial and lateral condyles of the humerus, greater tuberosity of the humerus, nuchal crests (all of them on both sides, right and left) and manubriosternal joint. Finally, the numerous cervical, thoracic and lumbar spinous processes are aggregated in three locations, and the 1–7th costochondral bilateral joints are aggregated in two locations (right and left).
- The SPARCC index evaluates 16 enthesis sites that predominantly include peripheral locations: the Achilles tendon, plantar fascia, greater trochanter, quadriceps tendon insertion into the patella, tibial tuberosity, medial and lateral epicondyles and supraspinatus insertion (i.e., greater tuberosity of the humerus) (all on both sides) [9].
- The LEI evaluates 6 peripheral locations: the Achilles tendon, medial femoral condyles and lateral epicondyles of the humerus (all right and left) [10].
- The MASES evaluates 13 enthesitis, both axial and peripheral: the Achilles tendon, the first costochondral joint, the seventh costochondral joint, posterior superior iliac spine, anterior superior iliac spine, iliac crest (all of them on both sides) and the fifth lumbar spinous process [11].

### Other variables collected

From the original ASAS-PerSpA study, we extracted data on the variables shown in Table 1. These included patient age and sex; diagnosis of axSpA, PsA or pSpA according to a rheumatologist; HLA-B27 status; extramusculoskeletal manifestations such as uveitis, psoriasis and inflammatory bowel disease; and peripheral musculoskeletal manifestations such as arthritis and dactylitis. Disease activity was evaluated by the Bath Ankylosing Spondylitis Disease Activity Index (BASDAI) and the Ankylosing Spondylitis Disease Activity Score-C reactive protein (ASDAS-CRP) [12, 13], while function was evaluated by the Bath Ankylosing Spondylitis Functional Index (BASFI) [14]. A concomitant diagnosis of the presence of secondary fibromyalgia according to a rheumatologist was also recorded, and the self-report Fibromyalgia Rapid Screening Tool (FiRST) was completed. Treatment information was also collected including the use of nonsteroidal anti-inflammatory drugs (NSAIDs), glucocorticoids, conventional synthetic (cs) drugs (methotrexate, leflunomide, sulfasalazine, hydroxychloroquine and azathioprine) and biological (b) disease-modifying antirheumatic drugs (DMARDs).

**Table 1:** Characteristics in the overall SpA population and in axSpA, pSpA and PsA patients

	Overall SpA population *N* = 4185	axSpA *N* = 2719	pSpA *N* = 433	PsA *N* = 1033
Age, mean (SD)	44.6 (13.8)	41.98 (13.0)	44.18 (14.4)	51.82 (13.0)
Sex male	2724 (61.0%)	1858 (68.3%)	203 (46.9%)	501 (48.5%)
Disease duration (SD)	14.50 (11.38)	14.35 (11.09)	10.06 (9.46)	16.75 (12.26)
HLAB27 positivity	2066/3120 (66.2%)	1709/2168 (78.8%)	197/316 (62.3%)	86/474 (18.1%)
Psoriasis confirmed by a dermatologist	1113/4464 (24.9%)	154/2718 (5.7%)	53/433 (12.2%)	894/1033 (86.5%)
Uveitis ever	738 (16.5%)	588 (21.6%)	75 (17.3%)	27 (2.6%)
Inflammatory bowel disease (IBD)	275 (6.15%)	132 (4.9%)	25 (5.7%)	6 (0.6%)
Peripheral joint disease ever (arthritis)	2541 (57%)	978 (36%)	410 (94.7%)	938 (90.8%)
Dactylitis	685 (15.3%)	164 (6.0%)	100 (23.1%)	382 (36.9%)
Fibromyalgia (rheumatologist’s opinion)	400 (9%)	212 (7.8%)	48 (11.0%)	120 (11.6%)
Fibromyalgia (FiRST)	775 (18.7%)	427 (17.2%)	69 (17.6%)	245 (24.9%)
BASDAI mean (SD)	3.8 (2.4)	3.7 (2.4)	4 (2.4)	4.3 (2.5)
BASFI mean (SD)	2.99 (2.6)	2.97 (2.6)	2.78 (2.6)	3.14 (2.7)
ASDAS-CRP mean (SD)	2.5 (1.14)	2.5 (1.1)	2.6 (1.2)	2.6 (1.1)
NSAIDs prescribed for any indication	3952/4185 (94.5%)	2574/2719 (94.7%)	423/433 (97.7%)	955/1033 (92.4%)
NSAIDs prescribed for enthesitis	1228/3952 (31.1%)	757/2574 (29.4%)	151/423 (35.7%)	320/955 (33.5%)
csDMARDs prescribed for any indication	2499/4185 (59.7%)	1182/2719 (43.5%)	370/433 (85.4%)	947/1033 (91.7%)
csDMARDs prescribed for enthesitis	665/2499 (26.6%)	326/1182 (27.5%)	101/370 (27.3%)	194/947 (20.4%)
bDMARDs prescribed for any indication	2177/4185 (52.1%)	1378/2719 (50.6%)	197/433 (45.5%)	602/1033 (58.3%)
bDMARDs prescribed for enthesitis	417/2177 (19.2%)	230/1378 (16.7%)	56/197 (28.4%)	131/602 (21.8%)

The results are expressed as the mean (standard deviation) for quantitative variables and absolute frequency (relative frequency) for qualitative values

*ASDAS *Ankylosing Spondylitis Disease Activity Score, *axSpA *axial spondyloarthritis, *BASDAI *Bath Ankylosing Spondylitis Disease Activity Index, *BASFI *Bath Ankylosing Spondylitis Functional Index, cs and *bDMARDs *Conventional synthetic and biological disease-modifying drugs, *FiRST *Fibromyalgia Rapid Screening Tool, *NSAID *Nonsteroid anti-inflammatory drugs, *PsA *Psoriatic arthritis, *pSpA *Peripheral spondyloarthritis, *SpA *Spondyloarthritis

All information was collected in a single interview by a study investigator or a research nurse.

### Statistical analysis

For the present study, only patients with a clinical diagnosis of axSpA, pSpA or PsA were included. Descriptive data are shown as the mean and standard deviation (SD) for quantitative variables and as absolute and relative frequencies for qualitative variables.

The most frequent locations of enthesitis across the three groups are described using absolute and relative frequencies.

The prevalence of patients with at least one enthesitis (at least one location with tenderness level > 1 on physical examination) was reported in the whole population as well as in each SpA subtype (axSpA, PsA and pSpA). These results were also depicted using Venn diagrams to evaluate which index identified more patients with enthesitis depending on the underlying disease. In addition, the global agreement of the different indices in the whole population and in the specific subgroups was evaluated with Fleiss’ kappa.

Finally, to determine which two indices yield similar prevalence rates in each SpA subtype, the pairwise agreement between indices was evaluated using Cohen’s kappa in the whole population and in the three groups.

All contrasts were two-sided and considered significant with a *p* value < 0.05. Data were collected, processed and analysed using RStudio 1.4.1106.

## Results

### Disease characteristics

A total of 4185 patients with a diagnosis of axSpA (2719, 60.9%), pSpA (433, 9.7%) and PsA (1033, 23.1%) according to the rheumatologist were included in this analysis. Among patients with axSpA (2719), 2137 (78.6%) had radiographic axSpA and 582 (21.4%) had non-radiographic axSpA.

Table 1 shows the most important characteristics of the study population. In the overall population, 61.0% were male, and the mean age was 44.4 (SD: 13.8) years. Males were more prevalent in the axSpA group, while females were more prevalent in the pSpA and PsA groups (53.1% and 51.5%, respectively). Fibromyalgia was observed in 9% of the overall population (axSpA: 7.8%, pSpA: 11.0% and PsA: 11.6%) according to the rheumatologist. Use of the FiRST questionnaire (a screening tool for fibromyalgia) indicated an increased prevalence of 18.7% in the overall population (axSpA: 17.2%, pSpA: 17.6%, and PsA: 24.9%).

### Enthesitis locations

**Fig. 1:**
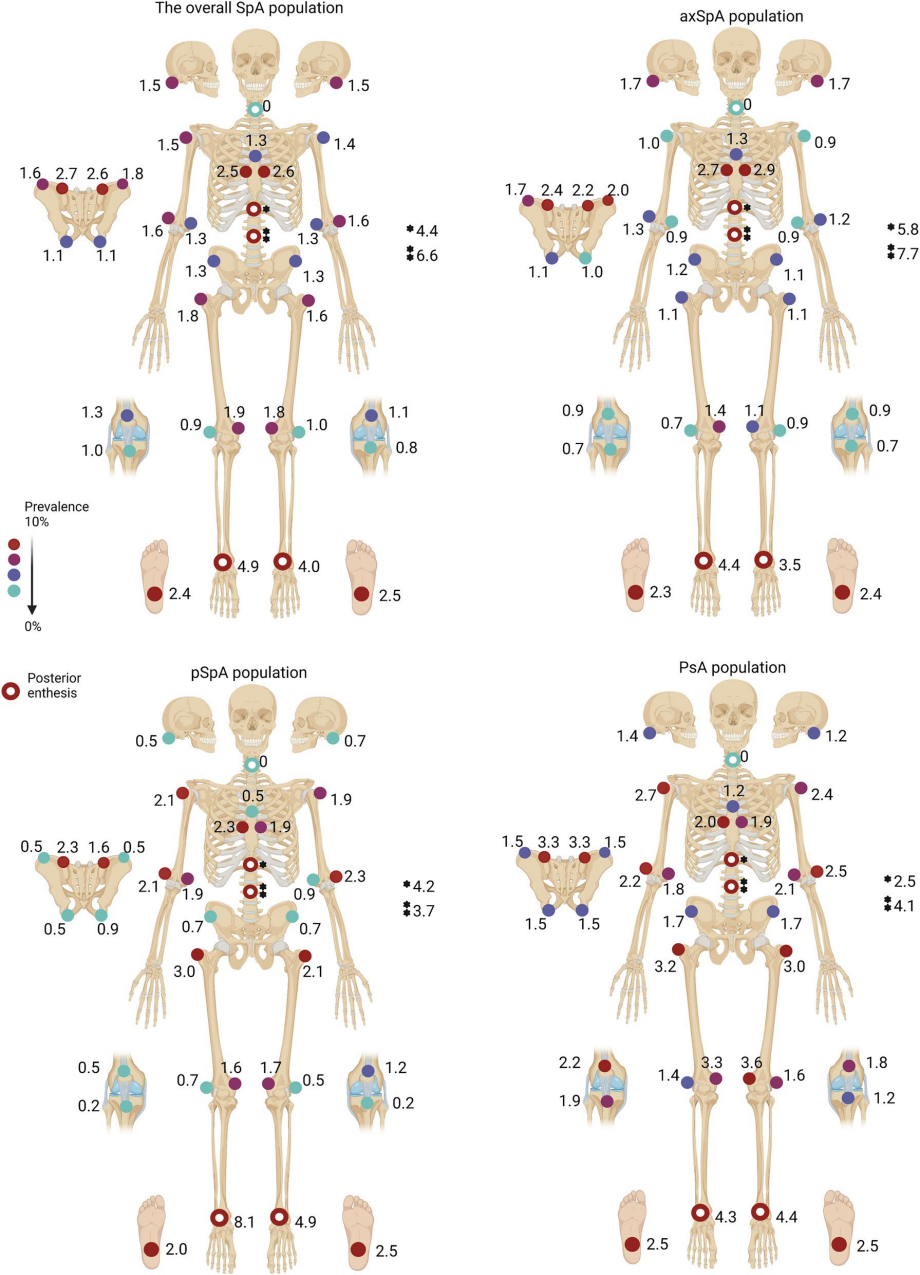
Individual locations of enthesitis in the overall SpA population, axSpA, pSpA and PsA The results are expressed as absolute frequency (relative frequency). axSpA, axial spondyloarthritis; PsA, psoriatic arthritis; pSpA, peripheral spondyloarthritis; SpA, spondyloarthritis

Figure 1 shows the individual locations of enthesitis in the SpA population as well as in the axSpA, pSpA and PsA groups. The most prevalent enthesitis locations in the SpA population were the lumbar spinous processes (6.6%), the insertion of the Achilles tendon (right) (4.9%), the thoracic spinous processes (4.4%), and the insertion of the Achilles tendon (left) (4.0%).

According to the anatomical region, we found that 7.7% of patients had enthesitis in the spinal region. We observed a prevalence of 3.3% in the anterior chest and 5.0% in the pelvis. Concerning peripheral locations, 3.7% of patients had enthesitis in the upper limbs, while 10.3% had enthesitis in the lower limbs.

Among patients with axSpA, similar results were found. The most common locations were the lumbar spinous processes (7.7%), the thoracic spinous processes (5.8%), and the insertion of the Achilles tendon, right (4.4%) and left (3.5%). In patients with pSpA and PsA, we observed different results: in pSpA patients, the insertion of the Achilles tendon, right (8.1%) and left (4.9%); the thoracic spinous processes (4.2%); and the lumbar spinous processes (3.7%) were the most frequent locations. In PsA patients, the insertion of the Achilles tendon, left (4.4%) and right (4.3%); the lumbar spinous processes (4.1%); and the medial condyle of the femur, left (3.6%), were the most frequent locations.

### Which index identifies the most patients with enthesitis?

In the overall population, 17.2%, 13.5%, 10.7%, and 8.3% of patients showed at least one enthesitis according to the MEI, MASES, SPARCC index and LEI, respectively.

Among patients with axSpA, at least one enthesitis was observed in 16.3%, 13.6%, 8.4%, and 6.7% according to the MEI, MASES, SPARCC index, and LEI, respectively. In patients with pSpA, the MEI identified 18.0% of patients with at least one enthesitis, the MASES identified 13.8% of patients, the SPARCC index identified 15.2% of patients, and the LEI identified 12.2% of patients. Finally, among patients with PsA, at least one enthesitis was observed in 17.2%, 13.9%, 11.4%, and 10.3% according to the MEI, SPARCC index, MASES, and LEI.

**Fig. 2:**
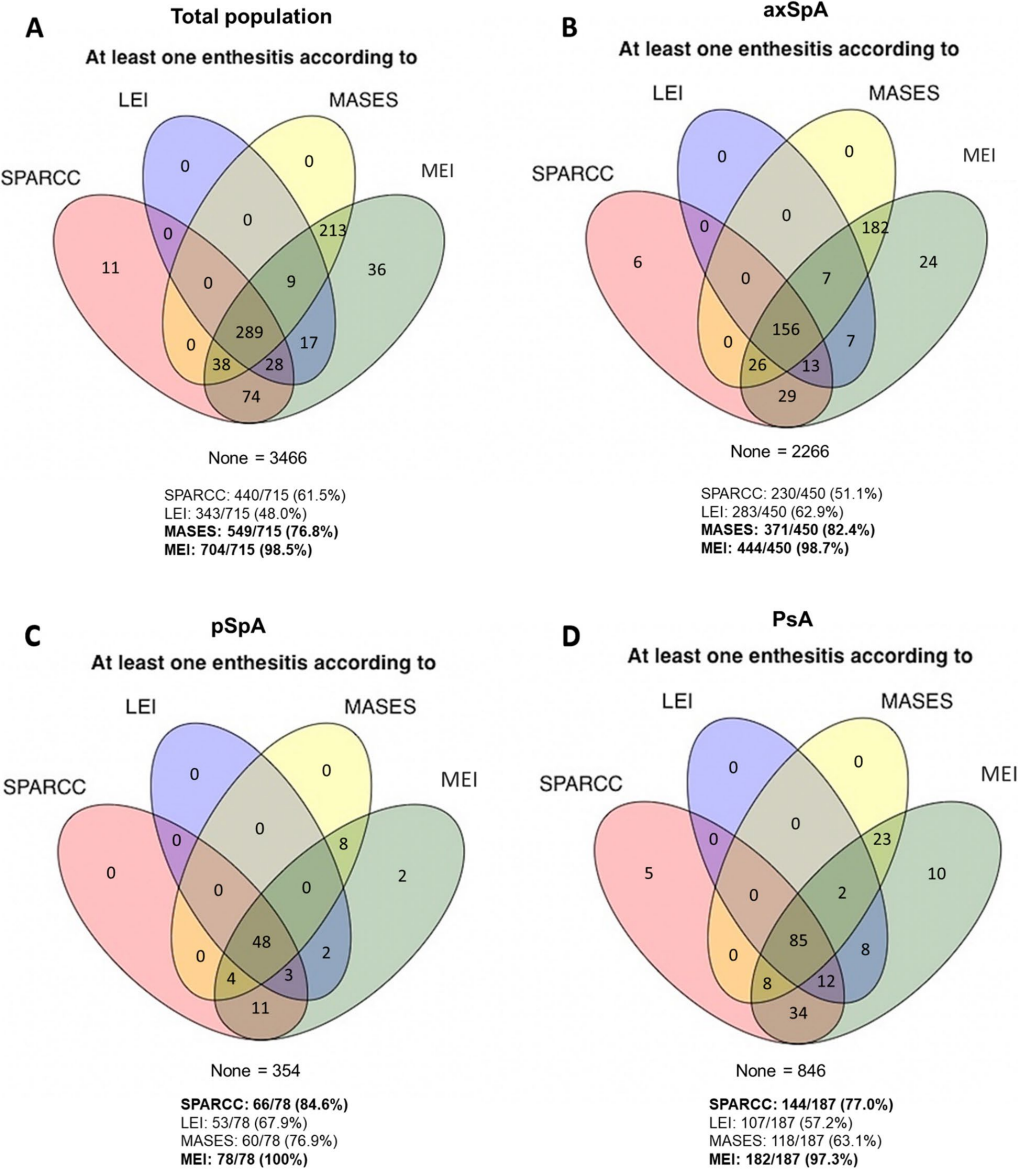
Prevalence of patients with at least one enthesitis captured by different indices MEI, Mander Enthesitis Index, SPARCC, Spondyloarthritis Research Consortium of Canada Enthesitis LEI, Leeds Enthesitis Index; MASES, Maastricht Ankylosing Spondylitis Enthesitis Score

Figure 2 shows the percentage of patients with at least one enthesitis according to the indices. In the total population, the MEI identified 98.5% of patients with at least one enthesitis. The MASES identified 76.8% of patients, while the SPARCC index identified 61.5% of patients. Finally, the LEI identified 48.0% of patients with at least one enthesitis. Similar results were found when separating the three SpA types. Among axSpA patients, the MEI identified 98.7% of patients with at least one enthesitis, the MASES identified 82.4% of patients, the LEI identified 62.9% and the SPARCC index identified 51.1%. Among patients with pSpA, the MEI identified the most patients with at least one enthesitis, followed by the SPARCC index, the MASES and the LEI (100%, 84.6%, 76.9% and 67.9%, respectively). Among patients with PsA, the MEI was the best index for identifying patients with at least one enthesitis (97.3%), followed by the SPARCC index (77.0%), the MASES (63.1%), and the LEI (57.2%).

### Agreement between indices

As observed in Table 2, in the total population, the MASES and MEI showed the strongest agreement for patients with at least one enthesitis (absolute agreement: 96.3%; Cohen’s kappa: 0.86, 95% CI: 0.83–0.88; *p* value: 0.001). The remaining pairwise comparisons also showed substantial agreement. The LEI vs. SPARCC index showed an agreement of 96.4% (Cohen’s kappa: 0.79, 95% CI: 0.76–0.82), while the MEI vs. SPARCC index had an agreement of 93.2% (Cohen’s kappa: 0.71, 95% CI: 0.68–0.74). The MASES vs. LEI, MASES vs. SPARCC index, and MEI vs. LEI also showed significant agreement but with lower kappa values (Table 2).

**Table 2:** Agreement between indices in the SpA population, axSpA, pSpA and PsA

Indices		Total *N* = 4185	axSpA *N* = 2719	pSpA *N* = 433	PsA *N* = 1033
LEI vs. SPARCC vs. MASES vs. MEI	Fleiss’ kappa:	0.705 (CI: 0.69–0.71; *p* value: 0.001)	0.663 (CI: 0.64–0.67; *p* value: 0.001)	0.83 (CI: 0.79–0.86; *p* value: 0.001)	0.74 (CI: 0.71–0.76; *p* value: 0.001)
LEI vs. SPARCC	Agreement:	96.4%	97.2%	96.1%	94.5%
	Cohen’s kappa:	0.79 (CI: 0.76–0.82;* p* value: 0.001)	0.80 (CI: 0.76–0.85;* p* value: 0.001)	0.84 (CI: 0.76–0.91; *p* value: 0.001)	0.74 (CI: 0.68–0.81; *p* value: 0.001)
MASES vs. SPARCC	Agreement:	92.0%	91.3%	94.9%	92.6%
	Cohen’s kappa:	0.62 (CI: 0.58–0.65;* p* value: 0.001)	0.56 (CI: 0.51–0.61; *p* value: 0.001)	0.80 (CI: 0.71–0.88; *p* value: 0.001)	0.67 (CI: 0.6–0.74; *p* value: 0.001)
MEI vs. SPARCC	Agreement:	93.2%	91.7%	97.2%	95.4%
	Cohen’s kappa:	0.71 (CI: 0.68–0.74; *p* value: 0.001)	0.62 (CI: 0.58–0.67; *p* value: 0.001)	0.90 (CI: 0.84–0.96; *p* value: 0.001)	0.83 (CI: 0.78–0.87; *p* value: 0.001)
MASES vs. LEI	Agreement:	92.9%	91.6%	96.1%	95.1%
	Cohen’s kappa:	0.63 (CI: 0.59–0.67; *p* value: 0.001)	0.55 (CI: 0.5–0.6; *p* value: 0.001)	0.83 (CI: 0.75–0.91; *p* value: 0.001)	0.75 (CI: 0.68–0.81; *p* value: 0.001)
MEI vs. LEI	Agreement:	91.4%	90.4%	94.2%	92.7%
	Cohen’s kappa:	0.61 (CI: 0.58–0.65; *p* value: 0.001)	0.54 (CI: 0.49–0.59; *p* value: 0.001)	0.78 (CI: 0.69–0.86; *p* value: 0.001)	0.70 (0.64–0.76; *p* value: 0.001)
MEI vs. MASES	Agreement:	96.3%	97.3%	95.8%	93.8%
	Cohen’s kappa:	0.86 (CI: 0.83–0.88; *p* value: 0.001)	0.90 (CI: 0.87–0.92; *p* value: 0.001)	0.85 (CI: 0.78–0.91; *p* value: 0.001)	0.75 (CI: 0.70–0.81; *p* value: 0.001)

*CI *Confidence interval, *LEI *Leeds Enthesitis Index, *MASES *Maastricht Ankylosing Spondylitis Enthesitis Score, *MEI *Mander Enthesitis Index, *SPARCC *Spondyloarthritis Research Consortium of Canada Enthesitis

In patients with axSpA, the MEI vs. MASES showed almost perfect agreement (97.3%; kappa: 0.90, 95% CI: 0.87–0.92), and the LEI vs. SPARCC index substantially agreed (97.2%; kappa: 0.80, 95% CI: 0.76–0.85), as did the MEI vs. SPARCC index (91.7%; kappa: 0.62, 95% CI: 0.58–0.67). The rest of the comparisons, such as the MASES vs. SPARCC index, MASES vs. LEI, and MEI vs. LEI, showed moderate agreement.

In pSpA patients, the SPARCC index vs. MEI showed the strongest agreement (97.2%; kappa: 0.90, 95% CI: 0.84–0.96). The agreement between the MEI vs. MASES (95.8%; kappa: 0.85, 95% CI: 0.78–0.91), the SPARCC index vs. LEI (96.1%; kappa: 0.84, 95% CI: 0.76–0.91) and the MASES vs. LEI (96.1%; kappa: 0.83, 95% CI: 0.75–0.91) was strong. Substantial agreement was observed between the SPARCC index vs. MASES and between the MEI vs. LEI.

Finally, among PsA patients, the MEI vs. SPARCC index showed the best agreement (95.4%; kappa: 0.83, 95% CI: 0.78–0.87), while the MEI vs. MASES (93.8%; kappa: 0.75, 95% CI: 0.70–0.81), LEI vs. MASES (95.1%; kappa: 0.75, 95% CI: 0.68–0.81) and LEI vs. SPARCC index (94.5%; 0.74, 95% CI: 0.68–0.81) showed substantial agreement. The MEI vs. LEI as well as the MASES vs. SPARCC index showed significant agreement but with lower kappa values.

## Discussion

This is the first study to evaluate the number of patients with enthesitis identified by the existing enthesitis indices in the whole spectrum of SpA. To date, these indices have been evaluated in specific populations, such as the axSpA or PsA populations, but no study has evaluated the performance of the four different indices in a single dataset. In this study, conducted with data from the large-sample ASAS-PerSpA study, we found that the prevalence of patients with at least one enthesitis differed according to SpA sutype and enthesitis index used. The use of a specific index may lead to the detection of a higher or lower number of enthesitis cases depending on the underlying disease.

In this study, enthesitis was originally evaluated through the MEI, which includes all possible enthesitis locations. We observed that in the whole SpA population as well as among axial SpA patients, the most common locations of enthesitis were the lumbar spinous processes, the thoracic spinous processes, and the insertion of the Achilles tendon (in that order). However, a previous study by AJ Mathew et al. reported that the most prevalent enthesitis locations in the axSpA population were the Achilles tendon, greater trochanter and plantar fascia, with no mention of lumbar enthesitis [15]. This can be explained by the use of the SPARCC index in that study, as that index does not include axial locations. Among the PsA population, as described in the previous study, the most prevalent locations of enthesitis were the Achilles tendon, the lateral epicondyle and plantar fascia enthesis. In another study that also used the SPARCC index, the three most frequent enthesitis locations were the Achilles tendon insertion, the plantar fascia, and lateral epicondyles [16]. In our study, the most prevalent locations in patients with PsA were the insertion of the Achilles tendon, the lumbar spinous processes and the medial condyle of the femur. As in the literature, the Achilles tendon was among the most prevalent locations of enthesitis. These differences might be explained by the MEI, which encompasses locations beyond those assessed by the SPARCC index, such as the lumbar spinous processes, the thoracic spinous processes and the medial condyle of the femur.

In our study, we applied the SPARCC index, LEI, MASES and MEI in the same population. These indices have previously been used separately (as in the two studies mentioned above); studies have also compared two or three of them, but not all four indices. Regarding the use of these different indices, the MEI identified the most patients with enthesitis in the overall population, possibly due to the vast number of locations evaluated. The index that identified the second highest number of patients was the MASES, which is derived from the MEI. The remaining two indices, the SPARCC index and LEI, identified a lower number of patients with enthesitis. However, these four indices exhibited different patterns among the three SpA subtypes.

In recent clinical trials of patients with axSpA, the LEI, MASES and SPARCC index have been used to evaluate clinical enthesitis. In an axSpA clinical trial on the efficacy and safety of secukinumab vs. placebo on Achilles tendon enthesitis, the authors used the LEI to evaluate the resolution of enthesitis [17]. In another study on the efficacy of etanercept for patients with active axSpA and enthesitis, the authors used the MASES and SPARCC index [18]. In a third study on the effectiveness of adalimumab in treating patients with axSpA with enthesitis and peripheral arthritis, the authors used the MASES [19], confirming the lack of consensus regarding the use of these indices in axSpA. Recently, the MEI has not been used in axSpA clinical trials, possibly because of the large number of locations and the time required for its evaluation. When we searched for clinical trials on patients with PsA, we found that the LEI and MASES were more commonly used to evaluate enthesitis. The LEI was used in two post hoc analyses that aimed to evaluate the resolution of enthesitis: one evaluating the efficacy of guselkumab [20] and a second evaluating ixekizumab [21]. In another study that aimed to improve enthesitis with methotrexate, the LEI was used for evaluation [22]. On the other hand, the MASES has been used in trials focused on PsA. For example, in a clinical trial with apremilast monotherapy, the enthesitis response among patients with PsA was evaluated through this index [23]. Another clinical trial assessed the 5-year response to apremilast in patients with PsA [24]. This index has also been used to evaluate enthesitis in a study on the use of ustekinumab in PsA patients naive to antitumour necrosis factor agents [25]. Conversely, the SPARCC index and MEI have not been used in recent clinical trials on patients with PsA.

Little is known regarding the agreement among the four enthesitis indices. Differences in agreement among them might be explained by the different locations evaluated. The MEI and MASES are similar, evaluating peripheral and axial enthesis. This could explain the strong agreement between the two in the whole population and in the axSpA group. Regarding pSpA and PsA, the MEI and SPARCC index had better agreement. This might be explained by the largely peripheral areas affected by enthesitis in these diseases (the Achilles tendon) and the peripheral areas evaluated by the SPARCC index. The LEI detected fewer patients with at least one enthesitis. This could be explained by its evaluation of only six locations. The LEI may be easier to implement in clinical practice, but the other three indices are recommended for use.

These results may not have implications in clinical practice since these indices are not commonly used due to the lengthy nature of evaluation. However, these results could be useful in clinical trials and observational studies evaluating the evolution of enthesitis under currently available treatments. As stated before, time availability may be a concern in clinical practice. Thus, the MEI, which takes the longest to evaluate, may not be the recommended index. Instead, as we observed agreement between indices, the MASES or the SPARCC index (depending on the disease evaluated) are recommended. Use of these indices could be incorporated into the protocol for the first patient visit to provide a baseline evaluation. In future visits, depending on the disease burden or management with treatment, these indices could be determined again. In clinical trials on enthesitis, these two indices might be recommended, depending on the disease of interest. The MASES appeared best for assessing enthesis in SpA and axSpA, while the SPARCC index appeared best for assessing enthesitis in pSpA and PsA.

The limitations of the study are similar to those of the perSpA database, from which the data were extracted. A shared limitation is the number of patients with axSpA, which was larger than that of the other two diseases. Another limitation is the operator-dependent technique for enthesitis index evaluation. A further limitation might be the lack of confirmation of enthesitis with an objective technique such as ultrasound or MRI, as well as the potential impact of current treatment on entheseal involvement and pain. In addition, concomitant fibromyalgia might influence the results since a significant overlap between the tender points evaluated by the MASES and those in fibromyalgia was recently demonstrated [26].

The strengths of the study are the vast number of patients included in the perSpA database and the international coverage. Another strength is that this study is the first to evaluate the whole spectrum of SpA with the four clinical indices available.

## Conclusions

In summary, we found that the prevalence of patients with at least one enthesitis differed across the SpA subtypes and depended on the enthesitis index used: the MEI and MASES identified the most axSpA patients with enthesitis, while the MEI and SPARCC index identified the most pSpA and PsA patients with enthesitis. Conversely, the LEI may underestimate the prevalence of enthesitis in these patients. These results suggest that the prevalence of enthesitis across SpA types differs depending on the disease and the index used.

## Data Availability

Data are available on reasonable request. Researchers desiring to use data collected during the study should contact the first author of the main manuscript, who will send a study proposal template to be completed by the applicant. Thereafter, the steering committee of the ASAS-PerSpA study will determine whether approve the proposal and share the data.

## Abbreviations

95% CI: 95% Confidence interval
ASDAS-CRP: Ankylosing Spondylitis Disease Activity Score-C reactive protein
axSpA: Axial spondyloarthritis
BASDAI: Bath Ankylosing Spondylitis Disease Activity index
BASFI: Bath Ankylosing Spondylitis Functional Index
bDMARDs: Biological disease-modifying antirheumatic drugs
CRF: Case report form
csDMARDs: Conventional synthetic disease-modifying antirheumatic drugs
Juv-SpA: Juvenile spondyloarthritis
IBD: Inflammatory bowel disease
LEI: Leeds Enthesitis Index
MASES: Maastricht Ankylosing Spondylitis Enthesitis Score
MEI: Mander Enthesitis Index
NSAIDs: Nonsteroidal anti-inflammatory drugs
PI: Principal investigator
PsA: Psoriatic arthritis
pSpA: Peripheral spondyloarthritis
ReA: Reactive arthritis
SD: Standard deviation
SpA: Spondyloarthritis
SPARCC: Spondyloarthritis Research Consortium of Canada
